# Putting into Practice Domain-Linear Motif Interaction Predictions for Exploration of Protein Networks

**DOI:** 10.1371/journal.pone.0025376

**Published:** 2011-11-01

**Authors:** Katja Luck, Sadek Fournane, Bruno Kieffer, Murielle Masson, Yves Nominé, Gilles Travé

**Affiliations:** 1 Group Onco-Proteins, Institut de Recherche de l'Ecole de Biotechnologie de Strasbourg, 1, BP 10413, Illkirch, France; 2 Biomolecular NMR group, Institut de Génétique et de Biologie Moléculaire et Cellulaire, 1, BP 10413, Illkirch, France; University of Rome, Italy

## Abstract

PDZ domains recognise short sequence motifs at the extreme C-termini of proteins. A model based on microarray data has been recently published for predicting the binding preferences of PDZ domains to five residue long C-terminal sequences. Here we investigated the potential of this predictor for discovering novel protein interactions that involve PDZ domains. When tested on real negative data assembled from published literature, the predictor displayed a high false positive rate (FPR). We predicted and experimentally validated interactions between four PDZ domains derived from the human proteins MAGI1 and SCRIB and 19 peptides derived from human and viral C-termini of proteins. Measured binding intensities did not correlate with prediction scores, and the high FPR of the predictor was confirmed. Results indicate that limitations of the predictor may arise from an incomplete model definition and improper training of the model. Taking into account these limitations, we identified several novel putative interactions between PDZ domains of MAGI1 and SCRIB and the C-termini of the proteins FZD4, ARHGAP6, NET1, TANC1, GLUT7, MARCH3, MAS, ABC1, DLL1, TMEM215 and CYSLTR2. These proteins are localised to the membrane or suggested to act close to it and are often involved in G protein signalling. Furthermore, we showed that, while extension of minimal interacting domains or peptides toward tandem constructs or longer peptides never suppressed their ability to interact, the measured affinities and inferred specificity patterns often changed significantly. This suggests that if protein fragments interact, the full length proteins are also likely to interact, albeit possibly with altered affinities and specificities. Therefore, predictors dealing with protein fragments are promising tools for discovering protein interaction networks but their application to predict binding preferences within networks may be limited.

## Introduction

Many of the protein interactions that function in cellular regulation and signalling are mediated by linear motifs that bind to globular domains. Such interactions are often specific, yet transient and therefore of low affinity [1]. The efficient prediction of such interactions together with their experimental validation would enormously increase our understanding of the cellular system. The occurrence of specific types of globular domains in protein sequences can mostly be predicted with high accuracy [2] [3] and promising work on linear motif predictions are published [4] [5]. However, the correct prediction of which instance of a linear motif will bind to which instance of a type of globular domain, hence the specificity in domain - linear motif interactions, remains one of the hot topics in computational biology.

Approaches for predicting domain-linear motif interactions have very often focussed on PDZ-peptide interactions. PDZs are a very abundant class of globular domains with 267 occurrences in the human proteome [6]. Human proteins often contain several copies of PDZs (up to 13) in their sequence. PDZs bind with a well defined pocket to linear motifs that are mostly situated at the extreme C-termini of proteins. The last residue (referred to as position p0) in PDZ-binding motifs is usually Val or Leu. The third last peptide residue (position p-2) can be either Thr or Ser (class I), hydrophobic (class II), or Glu or Asp (class III), thereby defining three main categories of PDZ-binding motifs [7] [8]. 339 experimentally verified PDZ-peptide interactions are currently annotated in the PDZbase [9] and 212 PDZ structures are listed in the ADAN database [10] indicating that PDZs are very well experimentally studied.

PDZs are implicated in the regulation of cell polarity, cell adhesion and intercellular communication [11]. The PDZ-containing proteins MAGI1 (Membrane-associated guanylate kinase inverted 1) and SCRIB (human Scribble) are in the centre of this study. MAGI1, which has six PDZ domains, was found to be located to adherens and tight junctions in epithelial [12] and endothelial cells [13], where it seems to be involved in the maintenance of the junctions and in cell signal propagation. SCRIB, which has four PDZ domains, is known to be involved in the establishment of adherens [14] and tight junctions [15] as well as in the regulation of cell polarity and cell migration [16]. Some data indicate that deregulation of MAGI1 [17] or SCRIB [18] can promote cell proliferation and tumorigenesis. Interestingly, proteins from different viruses were shown to bind via their C-terminal sequences to MAGI1 or SCRIB and to interfere with their cellular functions for promoting viral replication [19] [20]. For instance, the oncoprotein E6 produced by the human papillomaviruses (HPV) responsible for cervical cancer contains a PDZ-binding motif, which interacts with PDZ domains of MAGI1 and SCRIB [21] [22]. Deletion of this motif in HPV16 E6 impaired its capacity to promote cancer in transgenic mice [23] indicating that binding of E6 to MAGI1 and SCRIB might be implicated in the development of cervical cancer. Therefore, it would be important to better understand the signalling pathways, such as those of cell growth and apoptosis, that are regulated by MAGI1 and SCRIB and that are disrupted upon infection with oncoviruses such as HPV.

Until recently, only specific case studies had been published on the specificity of PDZ-peptide interactions, and the iSPOT tool [24] was for a long time the only attempt to predict PDZ-peptide interactions on a broader scale. In 2007 and 2008, two groups published outstanding large-scale studies on PDZ interactions providing insights into PDZ interaction specificities and strategies for their prediction [25] [26] [27]. Tonikian *et al.* [25] applied phage display to determine the binding profiles of 28 *C. elegans* and 54 *H. sapiens* PDZ domains using 10 billion random peptides. Stiffler *et al.* [26] applied microarrays and fluorescence polarisation to measure binding affinities between 157 mouse PDZ domains and 217 mouse peptides. All interactions and non-interactions (absence of interactions) determined by Stiffler *et al.* were used by Chen *et al.* [27] as training data for a PDZ interaction predictor. The prediction model was defined using the structure of the 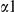-syntrophin PDZ domain bound to a seven residue-long peptide of which five are visible in the structure [28]. The model consists of 38 position pairs of domain and peptide residues that were seen to interact with each other in this particular structure. The training data was used in a Bayesian approach to obtain sub-scores for the occurrence of all possible combinations of amino acid pairs at these 38 position pairs. These sub-scores quantify the positive, neutral or negative contribution of a pair of amino acids at a certain position to the overall interaction between a PDZ domain and a peptide. The sum of the 38 sub-scores for a given PDZ-peptide pair represents the final score, which was suggested to indicate the binding strength of the potential interaction in question.

A very critical point for the development of protein interaction predictors is the availability of real negative interaction datasets [29]. Stiffler *et al.* [26] provide a negative PDZ interaction dataset, which has already been used to significantly improve PDZ interaction prediction quality [30] [31]. However, this negative dataset is the only one existing so far, which implies that PDZ interaction predictors trained with data of Stiffler *et al.* [26], such as the predictor of Chen *et al.* [27], cannot be tested on an independent negative dataset.

The numerous existing predictors for PDZ-peptide interaction specificities focus on the core PDZ domain or binding pocket of the PDZ and mostly on four or five residue long peptides [27] [30] [31] [32] [33] [34] [35]. Generally, it is assumed that interaction specificity predictions based on such protein fragments are also valid in the context of full length protein interactions and hence can be used to predict protein-protein interaction (PPI) networks. However, an increasing amount of biological studies on PDZ domains suggest that peptide residues upstream of the last five residues and domain residues outside of the binding pocket influence binding affinity and specificity [36] [37] [38] [39] [40]. Linker regions flanking the core PDZ domain as well as neighbouring domains, have also been found to influence binding [41] [42]. The term supramodule was introduced for neighbouring PDZs that are separated by particularly short linker sequences and that were shown to significantly influence each other's peptide binding (for a review see [43]).

Based on these observations, several questions are raised: First of all, how correct are PDZ interaction predictors in theory and in practice? Second, to which extent can specificity predictions based on protein fragments be transferred to full length proteins and how much influence do extensions of protein fragments have on affinity and specificity of the corresponding interaction? Third, can existing PDZ interaction predictors be used to extend our knowledge on PPI networks mediated by PDZ-peptide interactions? Here, we attempted to answer these questions by focussing on the well studied predictor published by Chen *et al.* [27]. First, we aimed at assessing its prediction quality *in silico* by using test datasets assembled by ourselves that consisted of real positive and negative interaction data for various PDZ domains. Then, by concentrating on PDZ domains of MAGI1 and SCRIB, we performed proteome-wide interaction predictions and experimentally validated a subset of those, allowing us to also assess the prediction quality *in vitro*. We also assessed how binding was influenced by extended protein fragments, i.e. peptides and PDZ constructs longer than those considered by the predictor. Finally, discovered interactors for MAGI1 and SCRIB were analysed with regard to new biological functions that can be linked to MAGI1 and SCRIB and that might be perturbed in tumours induced by oncoviruses or other factors. In total, this analysis allowed to highlight the power and limits of PPI network predictions involving PDZ domains, to uncover possible ways of improvements, and to obtain further insights into the mechanisms that define affinity and specificity of PDZ-peptide interactions.

## Results

### Development of real negative test datasets for benchmarking PDZ interaction predictors

We aimed at assessing the performance of the PDZ interaction predictor published by Chen *et al.* [27] with independent datasets of human PDZ-peptide interactions from low-throughput experimental studies. We assembled three test datasets (see Dataset S1) containing interactions and non-interactions involving 95 different human PDZ domains. The first test dataset contained 174 PDZ-ligand interactions including 109 human interactions from PDZbase [9] (a resource of experimentally verified PDZ-ligand interactions) plus 65 interactions that we manually collected from literature, mainly dealing with PDZ domains from MAGI1, 2 and 3. The PDZ domains from MAGI1, 2 and 3 are identical between human, mouse and rat when concentrating on the 16 domain amino acid positions used for predictions by Chen *et al.* Therefore, we included in the datasets interactions that we expect to occur between human proteins although they were originally described in the literature using rat and mouse PDZ domains.

**Figure 1:**
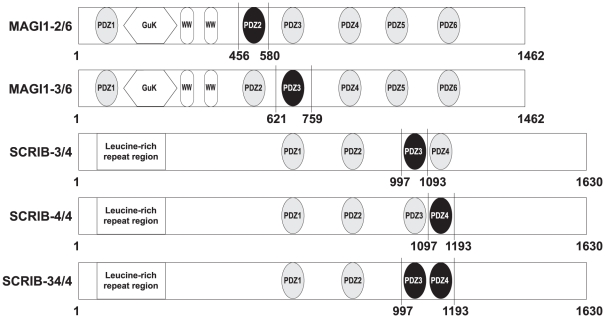
PDZ domains of MAGI1 and SCRIB. MAGI1 has 6 PDZ domains numbered from 1 to 6. SCRIB has 4 PDZ domains numbered from 1 to 4. The PDZ domains that were used for interaction measurements by SPR are highlighted in black and used domain boundaries are indicated.

The second and third test dataset contain negative interaction data that were assembled from published literature as follows. We took advantage of the particular characteristic of PDZ domains to occur as repeats within proteins (as illustrated in Figure 1). In order to experimentally determine the PDZ domain to which a peptide will bind out of the PDZ domains of a particular protein, each PDZ domain of the protein is tested separately for binding to the peptide. This approach usually yields one genuine interaction and many non-interactions. These non-interactions were annotated into one negative test set that in total contained 446 human non-interactions involving peptides bearing a PDZ-binding motif. The third test dataset contains 133 human non-interactions collected from the literature where the peptide has a disrupted PDZ-binding motif due to introduced mutations (substitutions or deletions). These real negative experimental data can be expected, as argued by Smialowski *et al.* [29], to outperform artificial negative data (such as randomised protein interactions) in terms of training and test performance.

### Benchmarking the PDZ-ligand interaction predictor of Chen *et al.*

When tested on the three established test datasets (Table 1) the predictor of Chen *et al.* obtained a sensitivity of 75.3% in agreement with that indicated by Chen *et al.* (76.5%) [27]. By contrast, the false positive rate (FPR) based on non-interactions with PDZ-binding motifs is about 48%, which is considerably higher than the FPR indicated by Chen *et al.* (24%). Furthermore, the FPR obtained for non-interactions without PDZ-binding motifs is about 26%, which represents a weak performance with regard to the relatively straightforward task to discriminate between peptides that bear a prototypical PDZ-binding motif or not. We then analysed separately, within our test datasets, the data involving human PDZ domains that are either orthologous or not orthologous to the mouse PDZ domains present in the training set of Chen *et al.* Sensitivity and FPR of these subsets show that the predictor tends to be over-optimistic for PDZ domains that are orthologous to domains present in the training data, and over-pessimistic for PDZ domains that are not orthologous to any domain present in the training data (third and fourth column in Table 1).

**Table 1:** Performance of predictor of Chen *et al.* for different test data sets.

	complete test data		training^a^		non-training^b^		MAGI1,2,3^c^	
sensitivity^d^	75.3%	(174)	90.7%	(97)	55.8%	(77)	65.9%	(41)
FPR PDZ^e^	48.2%	(446)	53.5%	(213)	43.3%	(233)	17.5%	(240)
FPR NoPDZ^f^	25.6%	(133)	27.6%	(58)	24.0%	(75)	4.0%	(50)

a test data containing only (non)-interactions with PDZ domains orthologous to those from the training data of Chen *et al.*

b test data containing only (non)-interactions with PDZ domains that were not orthologous to those in the training data of Chen *et al.*

c *t*est data containing only (non)-interactions with PDZ domains from MAGI1, 2 and 3 proteins. These subsets were analysed to verify that the overrepresentation of PDZ domains from these proteins did not introduce a bias in calculated sensitivity and specificities.

d percentage of interactions that were correctly predicted.

e percentage of non-interactions with PDZ-binding motif that were not correctly predicted.

f percentage of non-interactions without PDZ-binding motif that were not correctly predicted.

The numbers in brackets represent the total number of items in the respective test data set.

Our test datasets contain a large portion of interactions and non-interactions involving PDZ domains from MAGI1, 2 and 3. We separately calculated the sensitivity and FPRs of the predictor for subsets of the test datasets consisting only of PDZ domains of MAGI1, 2 and 3 (fifth column in Table 1). The results are considerably different from those obtained with the full datasets, indicating that the MAGI subset does over-influence the calculations.

### Prediction of natural PDZ-peptide interactions using the predictor of Chen *et al.*

The predictor of Chen *et al.* [27] was applied to PDZ domains of MAGI1 and SCRIB (see Figure 1 for the domain organisation of these proteins) with the aim of predicting, from the entire human proteome, natural interacting partners for these PDZs. For most domains, the numbers of predicted hits (proteins) were very high (Table 2, second column). An important proportion of these hits might be false positives in relation to the previously observed high FPR (Table 1). Indeed, one third of the C-terminal sequences of the returned hits had a non-hydrophobic amino acid at peptide position p0, in contradiction with most published literature concerning PDZ-binding sequence requirements. We analysed the amino acid composition of the pool of peptide sequences used to train the predictor of Chen *et al.* (Table S1) and observed that this pool of sequences had only V, L, I, F, C or A at position p0. This is due to the fact that the entire training pool of Chen *et al.* contained exclusively peptides that bound at least to one PDZ domain in the experiments of Stiffler *et al.* [26] and hence represent PDZ-binding sequences. In the training process, Chen *et al.* allocated zero (representing a neutral value) to all amino acids that were never seen at particular peptide positions. Whereas this strategy is sound when applying the predictor to peptides matching the general PDZ-binding consensus, it may lead to the selection of irrelevant peptides when querying an entire proteome. To take this issue into account, we applied an additional filter to accept only peptides ending with either C, Y, F, L, I, M, V, W or A, i.e. residues that were observed at position p0 in artificial or natural PDZ-binding peptides. This filter rejected 20 to 60% of the initial hits (Table 2, third column) and was systematically used further on in our study. Detailed information on the predicted interactions is provided in Dataset S2.

**Table 2:** Numbers of human proteins predicted to bind to PDZ domains of MAGI1 and SCRIB using the predictor of Chen *et al.*

PDZ domain	unfiltered hits	filtered hits^a^	num. prots. with highest score^b^	num. publ. binders^c^
MAGI1-1/6	0	0	0	1
MAGI1-2/6	457	300	93	4
MAGI1-3/6	160	107	0	1
MAGI1-4/6	43	30	0	3
MAGI1-5/6	1151	623	562	3
MAGI1-6/6	219	179	87	6
SCRIB-1/4	204	89	1	4
SCRIB-2/4	429	203	98	1
SCRIB-3/4	744	293	237	5
SCRIB-4/4	354	113	3	1

a proteins without residue C, Y, F, L, I, M, V, W or A at peptide position p0 were filtered out.

b numbers of proteins, which were predicted (after filtering) to bind to that domain and scored highest for that domain in comparison to the other domains.

c numbers of published mammal binders that we could identify from literature for each PDZ domain.

**Figure 2:**
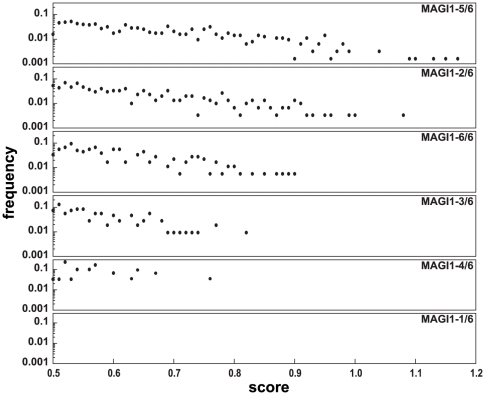
Score distribution of human C-terminal peptides predicted to bind to MAGI1 PDZ domains. Predictions were prefiltered for peptides having either C, Y, F, L, I, M, V, W or A at peptide position p0. Prediction scores were rounded to two decimal places and the frequencies of occurrence of scores within each interval were determined for each PDZ domain of MAGI1.

As shown in Table 2 (third column), some domains (e.g. MAGI1-5/6 - the fifth out of six PDZ domains of MAGI1) appeared to be very promiscuous as they had a very high number of hits, whereas others (e.g. MAGI1-4/6) had very few hits or even no hit at all (MAGI1-1/6). Within both MAGI1 and SCRIB, the PDZ domains obtaining the highest numbers of hits (MAGI1-5/6, 2/6 and 6/6, and SCRIB-2/4 and 3/4) were also the ones that obtained the highest scores (Table 2, fourth column). This might be correlated with our observation that scores obtained by different domains were distributed over different ranges (Figure 2). While investigating why some domains (e.g. MAGI1-5/6) showed higher scores and higher numbers of hits, we observed that particular peptide residues contributed very high subscores to the overall score for a domain-peptide pair. For instance, the occurrence of a Thr at position p-2 (a characteristic common to all class I PDZ-binding motifs) contributed a value of 0.64 to the prediction score for binding to MAGI1-5/6, while the overall value sufficient for a peptide to be classified as a hit by the preditor is 0.5. This means that any peptide possessing a Thr at position p-2 and residues at other positions that confer a predicted globally neutral effect for binding, would be classified as a binder for the MAGI1-5/6 domain. At present, we do not know whether this characteristic of MAGI1-5/6 is biologically meaningful or whether it just reflects some bias of the predictor's algorithm. Indeed, the predictions differ from published biological data (Table 2, fifth column), which indicate that the PDZ domain of MAGI1 attracting most binders is MAGI1-6/6, rather than MAGI1-5/6.

We also observed (Table S2) that numerous proteins were predicted to bind to more than one PDZ domain of MAGI1 or SCRIB, indicating that not only PDZ domains, but also C-terminal peptides, are considered to be promiscuous by the predictor. This may just originate from the lack of specificity of the predictor as already pointed out before in our analysis (see Table 1). However some PDZ-peptide interactions may indeed be really promiscuous and the predictor may be able to detect this trend.

### Structure-based analysis of domain amino acid positions implicated in peptide binding

**Figure 3:**
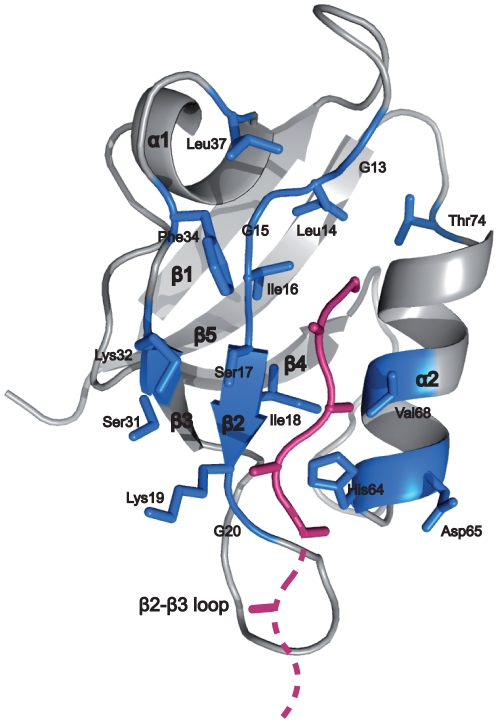
Structure of the PDZ domain of 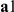-syntrophin used as reference by Chen *et al.* Residues coloured in blue represent the domain positions that are considered in the prediction model of Chen *et al.* The backbone and C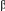 atoms of the bound peptide are represented as sticks in pink. The pink dashed line indicates where peptide residues upstream position p-4 would be situated in the structure. (PDBcode: 2PDZ).

**Figure 4:**
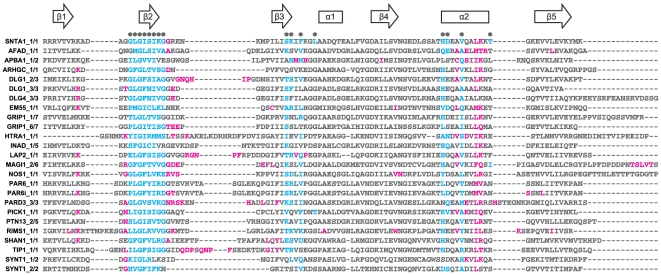
Atomic distance-based selection of peptide-contacting domain positions in different PDZ-peptide structures. For each PDZ domain of the alignment, we extracted from available structural data all domain residues that had at least one atom within a distance of 5 Å to bound peptide atoms. Blue letters indicate residues, which have been selected both, by Chen *et al.* and our approach. Red letters indicate residues, which have been selected by our approach but not by the model of Chen *et al.* Asterisks above the alignment indicate the PDZ residues chosen by Chen *et al.* to be close to peptide residues based on the structure 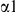-syntrophin (SNTA1, first line of alignment). Arrows and rectangles above the alignment indicate the positions of conserved 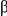-sheets and 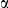-helices, respectively. Note that the sequence of the Par6 PDZ domain occurs twice in the alignment, corresponding to two different structures of Par6, one bound to an internal peptide, the other one bound to a regular C-terminal peptide.

In the prediction model of Chen *et al.* 16 domain and 5 peptide positions were selected for being implicated in specific binding of peptides to PDZs. This selection was based on one structure, α1-syntrophin [28] (Figure 3). The structural information on PDZs has considerably grown during the last years mainly due to structural genomics initiatives. Here, we comparatively analysed 42 structural complexes of 24 different PDZ domains to get a more general overview about amino acids involved in peptide recognition. Figure 4 shows that the set of domain amino acids found at less than 5 Å from the peptide in the various structures we analysed often differs from the set defined by Chen *et al.* in the structure of α1-syntrophin (these positions are indicated with asterisks above the alignment). For instance, domain positions Leu37 (α1 helix) and Thr74 ( α2–β5 loop) in α1-syntrophin (Figure 4), chosen by Chen *et al.*, were only selected once in the 23 other PDZ domains we analysed. Conversely, our approach (see Methods) selected more amino acids on α2 helix. In addition, while Chen *et al.* did not select any amino acid upstream of the GLGF-motif, our approach often selected residues in that region, especially a conserved positively charged position (Arg or Lys) within the 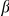
1-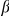
2 loop. The role of this amino acid for peptide binding is discussed in several studies [44] [45] [46]. Finally, our analysis often selected amino acids of the 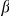
2-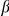
3 loop, whereas only one residue of that loop was selected in Chen *et al.*'s study. The selection of residues of the 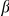
2-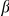
3 loop indicates that residues upstream position p-4 are proximal to this loop and therefore may also contribute to binding (Figure 3). Altogether, we suggest that more domain and peptide positions than those defined by Chen *et al.* may influence binding specificity.

### Experimental validation of predicted MAGI1-peptide and SCRIB-peptide interactions

From predictions obtained with the predictor of Chen *et al.* we selected 17 human and three viral peptides for interaction measurements against five PDZ constructs: the four single PDZ domains MAGI1-2/6, MAGI1-3/6, SCRIB-3/4, SCRIB-4/4, and the tandem construct SCRIB-34/4 (Figure 1). The 17 human peptides were selected based on different criteria: First, we selected peptides that were predicted to bind promiscuously to all four single PDZ domains. Second, we systematically included the two best predicted hits for each of the four PDZ domains. Third, we preferred proteins already shown to interact with PDZ domains. Further selection criteria were sequence diversity within the set of selected peptides and biological functions related to known functions of MAGI1 and SCRIB. These were inferred from Gene Ontology annotations (Ensembl v52 [47]) and information provided by UniProt [48]. The three viral peptides correspond to the C-terminus of HTLV1 Tax1, HPV16 E6, and a mutated form of HPV16 E6 (further on called 16E6L/V), where Leu at position p0 was mutated to Val. The latter peptide was already assayed against MAGI1 and SCRIB PDZ domains in previous SPR studies performed by our group, and therefore we used it as positive control for the present study. Table S3 provides detailed information about the 19 proteins.

For each of these 19 proteins two peptides were designed, *both of ten amino acids in length*. One peptide, called “long”, encompassed the last ten wild type residues of the protein (e.g. VMRLQSETSV for VANG2). The other peptide, called “short”, encompassed the last five wild type amino acids of the protein preceded by a GSGAG sequence (e.g. GSGAGSETSV for VANG2). This GSGAG sequence, composed of small neutral residues, was included to prevent the biotin tag N-terminally attached to the peptides to influence the binding to the PDZ domain. The “short” peptides, in which only the last five residues vary and correspond to natural proteins, would allow us to experimentally validate interaction predictions obtained with the predictor of Chen *et al.* that considers the last five residues in the prediction model. The long peptides (as well as the tandem PDZ construct) would allow us to address changes in binding affinity and specificity that might occur when using extended protein fragments.

**Figure 5:**
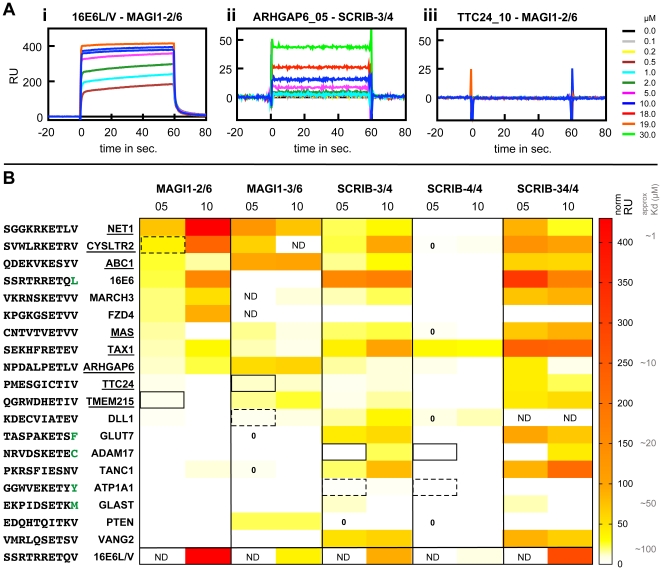
Overview of SPR experimental data. A: Representative sensorgrams for strong and weak interactions as well as non-interactions. An increase of the signal for injection of MBP-PDZ analyte is indicative of binding. (i) The higher the analyte concentration, the higher the 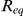 up to saturation, indicative of a specific interaction. (ii) For weak interactions the highest analyte concentration, which was injected due to device limitations, did not allow to reach saturation. (iii) Sensorgrams for non-interactions display no change in signal. B: Overview of measured RU signals and comparison to predictions. Normalised RU signals determined for a 10 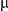
M concentration of MBP-PDZ were extracted from SPR sensorgrams and plotted as heatmap for 19 peptides in short and long versions *vs.* the five PDZ constructs MAGI1-2/6, MAGI1-3/6, SCRIB-3/4, SCRIB-4/4 and SCRIB-34/4. An approximate range of K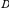 is indicated at the right side of the heatmap. 05 and 10 indicate short and long versions of peptides, respectively. ND = not determined. Signals of short peptides interacting with single PDZ constructs were compared to interaction predictions performed with the predictor of Chen *et al.* [27]. Rectangles and dashed rectangles indicate the first and second best hit for each PDZ domain, respectively, out of a proteome-wide screen. PDZ-peptide pairs that were predicted not to interact are labelled with zero. All other pairs of short peptides and single PDZ constructs were predicted to interact. Peptide names that are underlined indicate short peptides that were predicted and confirmed experimentally to bind to at least three of the four single PDZ domains. 16E6L/V served as control.

We opted for the surface plasmon resonance (SPR) method to measure these 190 (19 proteins×2 peptide versions×5 PDZ constructs) interactions. In SPR various concentrations of “analytes” (here, PDZ domains fused to the Maltose Binding Protein (MBP)) flow over surfaces presenting attached “ligands” (here, biotinylated peptides). The amount of analyte interacting with the ligand is measured and quantified in response units (RU). The intensity of this signal is proportional to the binding strength of the assayed interaction (Figure 5A). K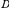 were obtained using a 1∶1 interaction model. However, these calculated K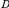 were rather inaccurate especially for weak interactions. Therefore, we preferred to rank the binding strengths of the 190 interactions using normalised RU signals at equilibrium (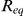) rather than K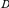 (see Methods for details). These normalised 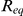 values were plotted in form of a heat map (Figure 5B). Table S4 contains experimental data for all SPR measurements performed in this study.

Nine out of nine published interactions (including 16E6L/V) were confirmed by our experimental data, of which three out of four published K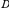 could be confirmed as well, all being high affinity interactions (see Table S3 for more details). This demonstrates the validity of our experimental SPR setup for testing PDZ-peptide interactions.

### Peptides do not bind as promiscuously as predicted to PDZ domains

Most tested peptides had been predicted to bind promiscuously to all four single PDZ domains (see Figure 5B, zeros indicate the very few PDZ-peptide pairs predicted not to interact). In practice, the peptides turned out to be much more selective than predicted. Only one peptide, TAX1 (derived from a viral protein), was found to interact with the four PDZ domains, and only at the condition of taking a very weak interaction into account. Even when we discarded the SCRIB-4/4 domain (which bound only one peptide as will be discussed later), we observed that, out of the 16 peptides predicted to bind the remaining three single PDZ domains, only 8 could be confirmed (see Figure 5B, underlined peptide names), again only at the expense of accepting very low interaction signals. This appears to confirm the high false positive rate of the predictor of Chen *et al.* that we have previously noticed (Table 1).

### The prediction scores do not correlate with interaction affinities

**Figure 6:**
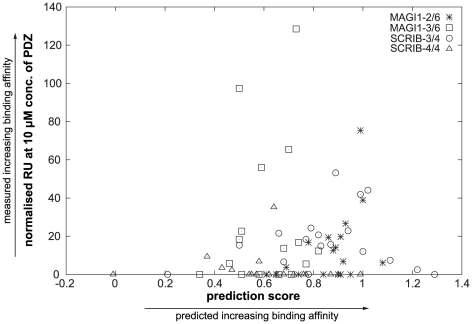
Comparing predicted to measured interaction intensities. The measured interaction intensities (in RU) between short versions of peptides and the PDZ domains MAGI1-2/6, MAGI1-3/6, SCRIB-3/4 and SCRIB-4/4 were plotted against the prediction scores obtained for the PDZ-peptide pairs with the predictor of Chen *et al.* The prediction scores did not correlate with measured signals. Note that SPR measurements were mostly performed for PDZ-peptide pairs that were predicted to bind to each other, explaining why the left region of the graph is empty.

Chen *et al.* have observed a correlation between prediction scores and binding affinities. In our set of data (19 short peptides vs. 4 single PDZ domains), we did not observe such correlation (for MAGI1-2/6 Pearson correlation coefficient r = 0.44 p-value = 0.07, for MAGI1-3/6 r = 0.13 p-value = 0.64, for SCRIB-3/4 r = 0.1 p-value = 0.69, for SCRIB-4/4 r = −0.08 p-value = 0.74) (Figure 6). In particular, the two best predicted hits for each PDZ domain turned out to be non-interactions or very weak interactions in all cases except one (Figure 5B, rectangles).

### SCRIB-4/4 may display very specific binding preferences

**Figure 7:**
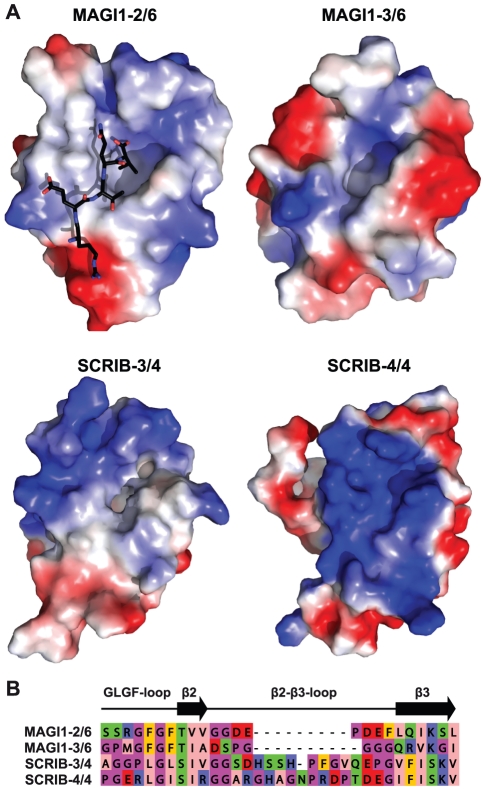
Structural particularities of SCRIB-4/4 in comparison to the three PDZ domains MAGI1-2/6, MAGI1-3/6, and SCRIB-3/4. A. The three experimental structures and one model (SCRIB-3/4) are shown in surface representation with red and blue indicating the electrostatic potentials. The structures are displayed in the same orientation as the PDZ domain in Figure 3. The peptide that was crystallised in complex with MAGI1-2/6 is shown in black. (PDB codes: 2I04, 3BPU, 1UJU for MAGI1-2/6, MAGI1-3/6, and SCRIB-4/4, respectively. The structure of SCRIB-3/4 was modelled from that of DLG4-1/3 (2KA9) using Modeller [79].) SCRIB-4/4 has a particularly positively charged surface around the peptide binding pocket in comparison to the other three domains. In addition, the pocket accommodating the hydrophobic residue at peptide position p0 is particularly shallow in SCRIB-4/4. These characteristics may explain the high ligand specificity displayed by SCRIB-4/4. B. Extract of the sequence alignment of the four PDZs illustrating differences within the GLGF-loop and the 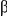
2-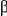
3 loop. SCRIB-4/4 presents a bulky R residue instead of a G in the GLGF-loop probably reducing the available space within the pocket.

SCRIB-4/4 was found to significantly bind to only one peptide, TAX1, despite of the fact that SCRIB-4/4 was predicted to bind to 15 out of the 19 peptides tested (Figure 5B). Remarkably, Zhang *et al.* [49] previously noticed that the SCRIB-4/4 domain did not bind any peptide in a phage display experiment. They interpreted this observation by suggesting that recombinant SCRIB-4/4 might be less stable than other PDZ domains. This possibility can be excluded, since we produced highly concentrated folded SCRIB-4/4 for NMR studies (data not shown), and the NMR structure of folded SCRIB-4/4 was solved by the RIKEN Structural Genomics Initiative (PDB code: 1UJU). We suggest that SCRIB-4/4 displays very specific peptide binding preferences, which can be inferred from analysis of available protein structures. We retrieved from the PDB the experimental structures of MAGI1-2/6, MAGI1-3/6 and SCRIB-4/4, and modelled the structure of SCRIB-3/4 (see Methods). The surface electrostatics representations of the four PDZ domains (Figure 7A) show that, in comparison to the other three PDZ domains, SCRIB-4/4 possesses many positive charges surrounding the peptide binding pocket. This should favour peptide sequences with negatively charged residues at position −1 and −3.

The “GLGF-loop”, which precedes the 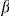
2 strand, coordinates the C-terminal carboxyl group of the peptide and also influences the width of the pocket accomodating the hydrophobic residue at p0 [45]. The first glycine of the “GLGF-loop” is replaced by a bulky arginine residue in SCRIB-4/4 (Figure 7B). This may sterically prevent binding of a peptide presenting a large hydrophobic side chain at p0 and might explain the shallow appearance of the pocket accommodating the peptide residue p0 (Figure 7A). These size and charge constraints may impose sequence properties only found in TAX1 (ETEV) out of the 19 peptides tested.

### Different preferences of PDZ domains for residues at peptide position p0

**Figure 8:**
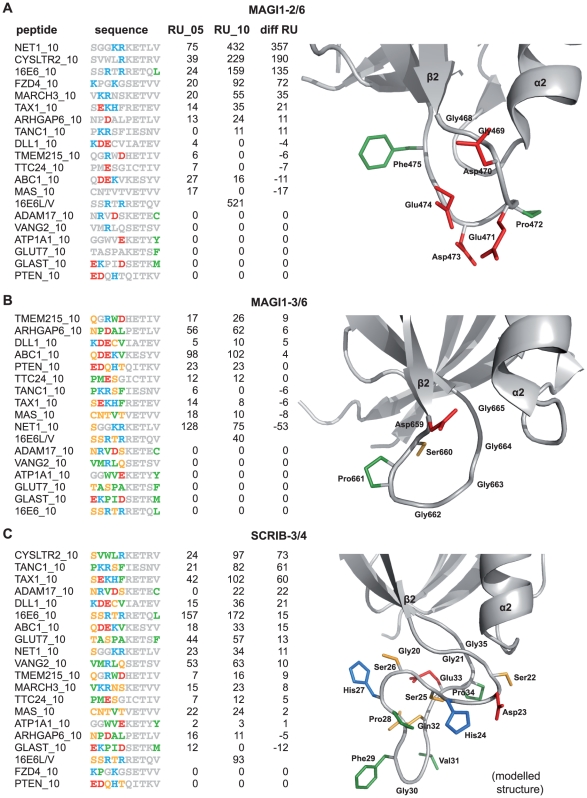
Influence of the 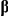
2-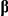
3 loop of PDZ domains on peptide binding. Columns indicate from left to right the names of the peptides, their sequences, the interaction intensities in RU for peptides with five and ten wildtype residues, and the interaction intensity difference between both. Peptides with five wildtype residues had the five N-terminal residues replaced with GSGAG. For each PDZ the part of the structure containing the 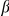
2-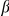
3 loop is shown with loop side chains represented as sticks. Amino acids in the sequences and structures are coloured as follows: red = negative charge, blue = positive charge, yellow = polar, green = hydrophobic. A. MAGI1-2/6 binds with increased affinity to peptides with positive charges upstream p-4 probably due to four negative charges in the loop (pdb code: 2I04). B. MAGI1-3/6 does not show any difference in affinity to short and long peptides, possibly due to four “neutral” glycines in the loop (pdb code: 3BPU). C. SCRIB-3/4 shows rather an unspecific increase in affinity for long peptides. The loop is very long and contains residues of all physico-chemical types.

Our interaction data reveal different binding preferences of the PDZ domains for specific hydrophobic amino acids at peptide position p0 (Figure 5B and Figure 8, see green residues at p0 in peptide sequences). SCRIB-3/4 seems to accept larger hydrophobic residues at p0 with a preference of leucine over valine. Indeed, SCRIB-3/4 binds stronger to wild type 16E6 as compared to the single mutant 16E6L/V, where the last residue of 16E6 has been mutated from leucine to valine. In contrast, MAGI1-2/6 binds stronger 16E6L/V than wild type 16E6, showing that MAGI1-2/6 preferentially accommodates valine in comparison to leucine. This was also observed by Thomas *et al.* [50] using full length E6 proteins. MAGI1-3/6 only accepts valine.

These different preferences for amino acids at p0 might be again correlated with amino acid variations in the conserved “GLGF-loop”. The alignment in Figure 7B shows that the two conserved hydrophobic positions of the “GLGF-loop” are occupied by phenylalanine residues in both MAGI1-2/6 and MAGI1-3/6 *vs.* two leucine residues in SCRIB-3/4. This might contribute to a wider pocket in SCRIB-3/4, explaining the preference of this domain for a C-terminal leucine in the bound peptide.

These different preferences for residues at p0 were only partially correctly predicted for MAGI1-2/6 and MAGI1-3/6 by the predictor of Chen *et al.* The predictor failed to predict these amino acid preferences for SCRIB-3/4 (see Dataset S2).

### Binding affinities and specificities change for extended interaction fragments

We observed that the tandem construct SCRIB-34/4 bound several peptides with higher affinity as compared to the single domain constructs SCRIB-3/4 and SCRIB-4/4 (Figure 5B). This increase seemed not to depend on the sequence of the peptides.

In addition, we observed that the long peptides often bound PDZ domains with different affinities as compared to the short peptides (Figure 5B). As highlighted in Figure 3, the additional wild type residues present in the long peptides, upstream position p-4, are likely to engage interactions with residues in the 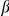
2-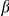
3 loop of the PDZ domains. Figure 8 shows part of the structures of the PDZ domains MAGI1-2/6, MAGI1-3/6 and SCRIB-3/4 comprising the region, where the 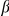
2-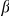
3 loop is situated (see Figure 7B for an alignment). Next to the structures, the differences in RU signals between long and short peptides are ranked from the greatest difference to the lowest. MAGI1-2/6 has four negatively charged residues in the 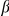
2-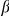
3 loop and shows strong increases in affinity for long peptides having positively charged residues at peptide positions upstream p-4. The closer these positively charged residues are positioned to p-4, the bigger is the increase in affinity for long versions of peptides. By contrast, negative charges at these peptide positions appear to be disadvantageous (Figure 8A). MAGI1-3/6 did not show significant differences in affinity and specificity between short and long peptides. This observation may be explained by the fact that the 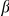
2-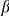
3 loop contains four consecutive glycine residues unlikely to influence peptide binding (Figure 8B). SCRIB-3/4 shows an unspecific increase in affinity for many long peptide versions. The 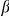
2-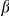
3 loop of SCRIB-3/4 is twice as long as for the other two PDZ domains and contains amino acids of diverse physico-chemical properties (Figure 8C). This loop might be able to adapt conformationally to many different sequences upstream of peptide position p-4, therefore providing advantageous contacts in most cases.

## Discussion

In this study we addressed the problem of predicting naturally occurring protein interactions mediated by PDZ domains and PDZ-binding peptides using the predictor of Chen *et al.* [27]. We analysed the predictor using theoretical and practical approaches. An important step for a fair assessment of prediction qualities is the application of real test datasets independent from the training data. To ensure this, we assembled a novel dataset of real negative PDZ-peptide interactions from the literature, which might turn out to be very useful for further development of PDZ interaction predictors.

Both the *in silico* and *in vitro* tests indicated that prediction accuracies were weak. We could demonstrate that the predictor of Chen *et al.* displays a high FPR, as recently suggested by Hui and Bader [30] and that predictions are biased towards the training interaction data. Prediction scores seemed not to correlate with interaction affinities, and amino acid preferences at peptide position p0 were only partially correctly predicted. These limitations may result from both an incomplete model definition and inadequate training of the model. Regarding model definition, we showed that PDZ domains display significant structural variation, so that the model of Chen *et al.*, which is based on a single PDZ-peptide structure, may have excluded residues that are important for peptide binding. Regarding model training, the interaction dataset of Stiffler *et al.* [26] provided values for only about one third of the vast number of the model's parameters (20×20×38 = 15200). The other two thirds of the parameters were given by default the value zero, assuming that they are neither positively nor negatively contributing to PDZ-peptide interaction affinities. This allowed in particular for the tolerance of disadvantageous amino acids or over-weighting of advantageous yet non-specific residues in peptides and PDZ domains. This problem was intensified by the fact that the negative training data only consisted of peptides that displayed PDZ-binding motifs limiting again the sequence space covered. To turn around these limitations, it might be relevant to reduce the number of parameters that have to be trained by grouping amino acids according to their various physico-chemical properties [51]. Additionally, a filter should be applied that removes all predicted interactions with very unlikely PDZ-binding sequences, as has been done in the present study.

The predictor of Chen *et al.* is based on minimal interacting fragments corresponding to single PDZ domains and five residue-long peptides. We investigated how extensions of these minimal fragments would influence binding. The peptides that showed binding to SCRIB-3/4 generally displayed an increase in binding affinity in the presence of the tandem construct SCRIB-34/4. Since the isolated SCRIB-4/4 domain hardly bound to any peptide, we hypothesise that SCRIB-4/4 contributed indirectly to the increase in affinity of the SCRIB-3/4 domain for its target peptides, maybe by stabilising its structure. Such a long range effect might be favoured by the fact that the linker sequence between the two domains is particularly short (around 10 residues). These observations indicate that SCRIB-34/4 may represent a supramodule as defined by Feng and Zhang [43]. In a recent structure-function study, we have also demonstrated that the affinity of the MAGI1-2/6 PDZ domain to its peptidic target is modulated by the sequence of the C-terminal flanking region of the core structure of the PDZ domain [41].

Analysis of structures of PDZ-peptide complexes from the PDB showed that peptide residues upstream of p-4 are proximal to the 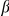
2-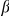
3 loop of PDZ domains, and SPR measurements showed that the same residues modulated binding. These observations confirm previous findings [36] [37] [38] [39] [40]. Moreover, we observed that the 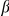
2-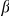
3 loop of different PDZ domains can display very different effects on affinity and specificity of peptide binding. The observation that flanking sequences surrounding a motif modulate its interactions with the target domain may also account for other classes of domain-peptide complexes [52].

Taken together, our results suggest that extensions of protein fragments may lead to changes in affinity and specificity. However, when comparing binding intensities obtained for long *versus* short peptide constructs or for single *versus* tandem PDZ domains, protein fragment extensions were never found to change an experimentally significant interaction into a non-interaction, nor vice-versa. Therefore, we hypothesise that whenever an interaction is detected between minimal fragments, it is likely that the full length proteins will also interact, albeit possibly with different affinities. Unfortunately, affinity measurements could not be undertaken with full length proteins to provide more evidence for this hypothesis due to experimental limitations in handling large proteins *in vitro*.

Our experimental data showed that many peptides bound weakly, with affinities much weaker than 20 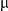
M, to several of the PDZ domains tested. These observations are consistent with results of Wiedemann *et al.* [53], who predicted that for a K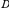 cutoff as low as 50 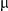
M, hundreds of ligands would bind to three distinct PDZ domains with largely overlapping specificity ranges. It is often stated that interactions stop to be biologically relevant when their affinity dissociation constants exceed a given threshold (e.g. 100 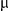
M). Such statements may have to be reconsidered when dealing with affinities determined from protein fragments, such as PDZ-peptide interactions, because as our data indicates, weak and promiscuous interactions might become stronger and more specific when moving from short protein fragments towards full length proteins.

Based on the results presented here we suggest FZD4, TMEM215 and ARHGAP6 as new interactors for MAGI1; TANC1, GLUT7, DLL1, MAS and NET1 as new interactors for SCRIB; and ABC1, MARCH3 and CYSLTR2 as new interactors for both MAGI1 and SCRIB. Remarkably, several of these proteins are proven or putative membrane proteins (FZD4, TMEM215, GLUT7, ABC1, MARCH3, MAS, CYSLTR2, DLL1) while the three remaining ones (ARHGAP6, TANC1, NET1) are involved in activities localised to the membrane. Indeed, SCRIB and MAGI1 were already known to localise to the membrane where they interact with numerous proteins involved in signal transmission, and more particularly in G protein mediated signalling. On the one hand, MAGI1 had been shown to interact with NET1 [54] [40], a guanine nucleotide exchange factor (GEF) specific for the small G protein RhoA, as well as with PDZ-GEF1 [55], another GEF specific for the small G proteins Rap1A, Rap1B and Rap2B. MAGI3, a close paralog of MAGI1, has been shown to interact with the G protein coupled receptors (GPCRs) FZD4 [56] and LPAR2 [57], and to interact with the integral membrane protein VANG2 leading to the activation of the JNK pathway via the small G protein Rac [56]. On the other hand, SCRIB had been found to interact with two GEFs, 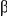
PIX [58] and ARHGEF16 [49], leading to activation of the small RhoA family G proteins Rac1 or Cdc42 [59] [60]. SCRIB has also been shown to interact with TSHR (a GPCR) [61].

**Figure 9:**
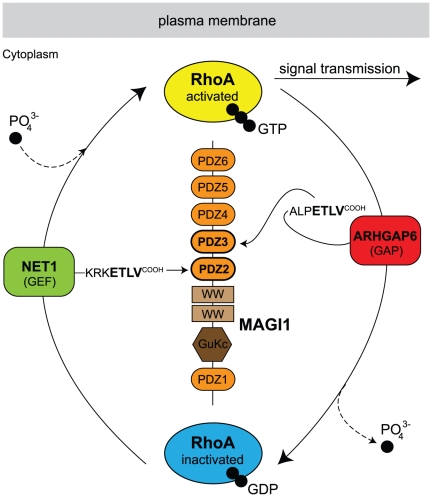
Suggested model for MAGI1 scaffolding function in Rho GTPase mediated signalling. Our data showed that PDZ2 and PDZ3 of MAGI1 bind preferentially to the C-termini of NET1 (green) and ARHGAP6 (red), respectively. NET1 is a guanine nucleotide exchange factor (GEF), which transfers a phosphate group (PO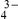) to the small GTPase RhoA, which in its GTP-bound form (yellow) is predominantly associated with the membrane and stimulates downstream signalling pathways. ARHGAP6 is a GTPase-activating protein (GAP), which induces RhoA to release a phosphate group, resulting in the shutdown of RhoA signalling. Inactivated GDP-bound RhoA (blue) is mostly present in the cytoplasm. This indicates that MAGI1 recruits, via two adjacent PDZ domains, one activator and one inhibitor of the RhoA signalling pathway. Remarkably, the four last residues of the two proteins NET1 and ARHGAP6 are identical, hence the distinct binding preferences of the two C-terminal peptides for PDZ2 and PDZ3 must be defined by residues upstream.

In line with these published findings, several of the novel putative interactors of MAGI1 and SCRIB that we identified are also involved in G protein signalling. FZD4, CYSLTR2 and MAS are GPCRs; NET1 is a GEF; ARHGAP6 is a GAP (G protein activating protein); ABC1 is a membrane transporter known to recruit two GEFs (PDZRhoGEF and LARG) involved in Cdc42 and RhoA signalling [62] [63]. Therefore, our data reinforce the view that MAGI1 and SCRIB act as scaffolds that assemble proteins close to membranes to regulate G protein signalling. A remarkable instance is MAGI1 which, as indicated by our data, might be able to recruit simultaneously, via neighbouring PDZ domains, a GEF (NET1) and a GAP (ARHGAP6) that are both specific for the small GTPase RhoA, while possessing inverse enzymatic activities (Figure 9).

MAGI1 and SCRIB are known to participate to the regulation of neuronal synapses via interaction with numerous proteins [64] [60] [65] [66]. Accordingly, TANC1, which was in our hands the strongest cellular binder of SCRIB, is a scaffold component protein in post-synaptic density regions [67]. Some other interactions suggested by our work seem to provide novel links between MAGI1 and SCRIB and pathways in which they were not yet known to participate: Wnt/JNK pathway regulation (FZD4), Notch pathway regulation (DLL1) [68], immune response (CYSLTR2) [69], iron uptake (MARCH3) [70], blood vessel regulation (MAS) [71], glucose transport (GLUT7) [72]. These new interactions can provide interesting starting points for exploration of potential new *in vivo* functions of MAGI1 and SCRIB that might be perturbed upon infection with HPV.

In this work, we showed that inferring protein interaction networks from predictions based on interacting protein fragments should involve at least two very distinct steps. The first step requires accurate prediction of interactions between the isolated protein fragments considered by the predictor. The predictor we used here for completing this step turned out to be rather inaccurate. There is much room for improving this step, in particular by integrating the wealth of structural information recently accumulated about protein domains, especially PDZs. The second step requires correct extrapolation of predicted fragment interactions to interactions between full length proteins. Our data indicate that such an extrapolation may be possible qualitatively, but not necessarily quantitatively. Therefore, while inferring protein interaction networks from minimal interacting fragment predictions appears as a reasonable perspective, more refined predictions addressing binding specificities in these networks remain a challenging, yet fascinating prospect.

## Materials and Methods

The programming and data analysis was done using python (www.python.org), biopython [73], gnuplot (www.gnuplot.info) and PyMOL (www.pymol.org). We used the same human proteome as described in Luck *et al.* [74] to perform the proteome-wide screens in this study.

### Prediction quality assessment

We assessed the performance of the predictor of Chen *et al.* [27] by applying the commonly used measures *Sensitivity* (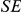) and *False Positive Rate* (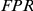) of the ROC analysis. Here, the sensitivity is defined as the percentage of PDZ-peptide interactions that were correctly predicted ( = True Positives (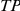)) and is calculated as follows:
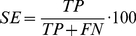
(1)where 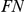 specifies the number of False Negatives (PDZ-peptide interactions not correctly predicted). The False Positive Rate is defined as the percentage of PDZ-peptide non-interactions that were *not* correctly predicted ( = False Positives (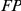)) and is calculated as follows:
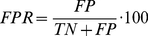
(2)where 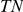 specifies the number of True Negatives (PDZ-peptide non-interactions correctly predicted).

### Implementation, test, and application of the predictor of Chen *et al.*

Chen *et al.* [27] trained the predictor in two different ways, called the binary and affinity mode, of which each of them can be used separately to apply the predictor. For the binary mode the predictor was trained without consideration of measured binding affinities (e.g. the training data was simply split into interactions and non-interactions). In the affinity mode, binding affinities were directly included in the training process. For all predictions performed in this study, the binary mode was used. No information about performance qualities was provided by Chen *et al.* for the affinity mode. We performed a comparison of both modes that revealed extremely different predictions with the binary mode providing more reliable results (data not shown). The predictor returns a score for each PDZ-peptide pair, which can be used to estimate the likeliness that the PDZ domain will bind the respective peptide. The higher the score, the more likely the interaction. Here, we used a score cutoff of 0.5, which should yield a sensitivity of 76% and FPR of 24% as specified by Chen *et al.*

Each of the 95 human PDZ domains in the test datasets were added to the alignment of mouse PDZ domains provided by Chen *et al.* in order to define the 16 amino acid positions on which predictions are based. Mafft [75] was used to obtain a preliminary alignment, which was corrected manually using Jalview [76] and structural information, if available. The alignment is provided in Dataset S3.

The training set containing 93 peptides of Chen *et al.* was not provided in the publication. The set of peptides from the training data was reconstructed as described by Chen *et al.* taking every peptide that was seen at least once in an interaction with a PDZ domain in the experimental data obtained by Stiffler *et al.* [26]. This revealed 108 peptides.

In Text S1 and Dataset S4 we provide guidelines and programming code, respectively, for users of the predictor of Chen *et al.*, who wish to follow our developed protocol.

### PDZ pocket analysis

Available structures of PDZ-ligand complexes were analysed in order to assess important domain residues for ligand recognition. A keyword search with “PDZ” in the PDB [77] revealed 267 structures. Crystal structures were excluded, if the PDB files did not contain coordinates of the full complex but just of one chain (e.g. PDB code 2EGN). After manual inspection, a final set of 42 structures with PDZ-peptide complexes was retained for further analysis representing 24 unique PDZ domains. For each PDZ domain all structural models obtained by NMR and all complexes shown in the crystal obtained by X-ray were taken into consideration for the determination of all domain residues that are in close proximity to bound peptides. A domain-peptide residue pair was only accepted, if in all complexes of this particular PDZ domain the distance between the two amino acids was in average below a defined threshold. Three different distance measures were implemented: C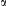 distances, distances between residue's centre of mass, and minimal atom distances between residues. Different thresholds were tested from 0 to 40 Å. The distance measure and cutoff that represented best the selection of the 16 domain amino acids in 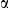-syntrophin of Chen *et al.* [27] was chosen: minimal atom distance with a threshold of 5 Å.

The PDZ sequences shown in Figure 4 were extracted from the following PDB entries and chains: SNTA1_1/1 (2PDZ A), AFAD_1/1 (2AIN A), APBA1_1/2 (1U38 A), ARHGC_1/1 (2OS6 A), DLG1_2/3 (2AWW A), DLG1_3/3 (2I0I C), DLG4_3/3 (1TP5 A), EM55_1/1 (2EJY A), GRIP1_1/7 (2QT5 A), GRIP1_6/7 (1N7F B), HTRA1_1/1 (2JOA A), INAD_1/5 (1IHJ A), LAP2_1/1 (1N7T A), MAGI1_2/6 (2KPL A), NOS1_1/1 (1B8Q A), PAR6_1/1 (1RZX A), PAR6i_1/1 (1X8S A), PARD3_3/3 (2K20 A), PICK1_1/1 (2PKU A), PTN13_2/5 (1D5G A), RIMS1_1/1 (1ZUB A), SHAN1_1/1 (1Q3P B), TIP1_1/1 (3DIW A), SYNT1_1/2 (1W9E A), SYNT1_2/2 (1V1T A).

### Structure modelling

The structure of the PDZ domain SCRIB-3/4 was modelled using the program Modeller 9v7. The structure template was obtained by querying the PDB with the sequence of SCRIB-3/4 (using the BLAST option) and choosing the structure with the best sequence match (PDZ domain DLG4-1/3, PDB-code 2KA9, 45% sequence identity, e-value 1.0E-11). Modeller was run using the automodel routine and default options. Model quality was assessed using the output information of Modeller and visual inspection. A model of SCRIB-3/4 of intermediate quality was sufficient for the purpose of this study.

### cDNA constructs

The cDNA encoding residues 448–572 and 613–752 of mouse MAGI-1 (UniProt acc.: Q6RHR9-1) encoding for MAGI1-2/6 (100% identical to human MAGI1-2/6) and MAGI1-3/6 (99% identical to human MAGI1-3/6) PDZ domains, respectively, were inserted into the NcoI/KpnI sites of the pETM-41 expression vector (EMBL) containing a 6×His-MBP tag followed by a TEV protease cleavage site. A similar cloning strategy was adopted for cDNA bearing residues 997–1093, 1097–1193 and 997–1193 of human SCRIB (Uniprot acc.: Q14160-1) encoding for SCRIB-3/4, SCRIB-4/4 PDZ domains and SCRIB-34/4 tandem PDZ construct, respectively.

### Protein sample production

Bacterial over-expression of PDZ domains was performed using BL21 DE3 *Escherichia coli* cells in 300 ml of M9 minimal medium supplemented with 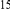
NH
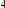
Cl at 37
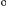
C until an OD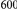 of 0.6 was reached. Cultures were then adjusted to 0.5 mM isopropyl-D-thio-galactopyranoside (IPTG) and transferred to 15

C overnight. Plasmid loss was suppressed by adding 15 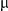
g/ml of kanamycin to the expression media. Expression cultures were harvested by centrifugation. The pellets were stored at −20
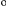
C.

### MBP-PDZ domains purification

Bacterial expression of 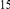
N-labeled 6×His-MBP-PDZ constructs were sonicated in buffer A (50 mM Tris-HCl at pH 6.8, 200 mM NaCl, 1 mM DTT) supplemented with 1 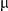
g/ml DNase I and RNase A and EDTA-free anti-protease cocktail inhibitor (Roche), cleared by ultracentrifugation at 60000

g and filtered (Millipore 0.22 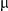
m). MBP-PDZ extracts were loaded on an amylose column (New England Biolabs) pre-equilibrated with buffer A. Protein was eluted with buffer A supplemented with 10 mM maltose. MBP-PDZ samples were then subjected to a 15 hour ultracentrifugation at 130000

g prior to loading on a Hiload 16/60 Superdex 75 gel-filtration column (Amersham Biosciences) pre-equilibrated with buffer B (20 mM sodium phosphate at pH 6.8, 200 mM NaCl) resulting in pure and mono-disperse protein samples according to the column calibration. The concentration of purified MBP-PDZ fusion samples was evaluated from UV absorption measurements at 280 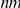. After SPR experiments MBP-PDZ fusions were cleaved by TEV and PDZ domains were separated from MBP by gel size exclusion chromatography. Subsequently, 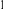
H-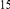
N heteronuclear single quantum coherence (HSQC) spectra were recorded on a 600 MHz Bruker instrument in order to verify structural integrity of the domains.

### Synthetic peptides

The synthetic peptide 16E6L/V (RSSRTRRETQV), corresponding to the last 11 C-terminal residues of HPV16 E6 with the last residue L mutated to V, was synthesised by the Chemical Peptide Synthesis Service, IGBMC, France. Lyophilised peptide was re-suspended in water, passed on a NAP-5 desalting column (GE Healthcare) in order to remove residual contaminants. The desalted peptide was lyophilised prior to its dilution into buffer A. The peptide was checked by homonuclear 2D NMR experiments and its concentration estimated to be at 6 mM by measuring the peptide bond absorption at 205 nm as described previously [40]. All other synthetic peptides with biotin at N-terminus that were used as ligand in surface plasmon resonance experiments were synthesised by JPT Peptide Technologies GmbH, Berlin, Germany. Lyophilised peptides were re-suspended in water at a final concentration at 10 mM. The pH of peptide solution was adjusted to 6.8.

### Surface plasmon resonance (SPR) measurements

Data were collected on a Biacore 2000 instrument (Biacore AB/GE Healthcare Bio-Sciences Corp., Piscataway, NJ, USA) at 25
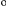
C. SPR experiments (ligand immobilisation and binding measurements) have been performed as described in Fournane *et al.* [40]. Briefly, biotinylated peptides (instead of GST-fused recombinant peptides) were immobilised on CM5 sensorchips on which Neutravidin was previously attached. The MBP-PDZ domain analyte was injected at 8 to 10 different concentrations ranging from 0 up to 30 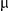
M. Data were processed using the BiaEvaluation 3.2 software (Biacore AB/GE Healthcare Bio-Sciences Corp.) using “double referencing” [78] in which sensorgrams were corrected for buffer effects and bulk refractive index changes. Representative sensorgrams are shown in Figure 5A.

The steady-state binding signal (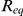) was derived by averaging the signals in a five second window at equilibrium. Steady-state analysis was performed by fitting the average signal 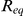 as a function of total MBP-PDZ concentrations, assuming a simple 1∶1 interaction binding isotherm model. For many weak interactions we observed calculated binding affinities (K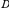) with fits that produced high 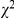 suggesting that the K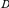 were likely to be inaccurate (see Table S4). Reasons for this inaccuracy are likely to be the following: 1. As previously described [40], several repetitions of all the measurements are required to determine accurate K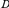. In our case, such repetitions were not achievable in reasonable time due to the large amount of interactions measured in this study. 2. The highest injected analyte concentration restricts the maximal K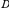 (weakest interaction) that can be accurately obtained. 3. A K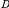 is estimated based on a mathematical extrapolation of observed 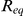 signals leading to additional uncertainty. Based on these reasons, we considered the calculated K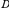 not as accurate enough to be used for absolute binding strength comparison in this study. We rather performed a relative analysis of binding strengths using directly 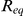 signals which are not biased by any mathematical assumption. We focussed on 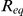 signals obtained at 10 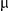
M MBP-PDZ concentration, which have been systematically measured in duplicate. The 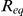 signal is directly proportional to the molecular weight of the analyte and the amount of immobilised ligand. Therefore, the 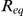 signals were normalised taking those into account before being used for binding strength comparison. The large amount of raw experimental data, which have been collected and the methodological approach that we have developed for their exploitation will be presented and discussed in detail in a separate, SPR-oriented paper.

## Supporting Information

Dataset S1 **PDZ interaction and non-interaction test datasets.** The archive contains three files, one for each test dataset established: interactions, non-interactions with PDZ-binding motif, and non-interactions without PDZ-binding motif. First column: PDZ domain, second column: name of binder, third column: C-terminus of binder.
(BZ2)

Dataset S2 **Prediction results of proteome-wide screen for MAGI1 and SCRIB PDZ-binding ligands using the predictor of Chen** ***et al.*** **** [**27**] **.** The prediction results were performed in binary mode using a cutoff of 0.5 and are provided without any additional filtering. No result file is provided for the PDZ domain MAGI1-1/6 because the screen did not reveal any peptides for this domain.
(BZ2)

Dataset S3 **Alignment of human PDZ domains.** The archive contains an alignment in fasta format of 95 PDZ domains. These include all PDZ domains that occur in the three test datasets as well as all MAGI1 and SCRIB PDZ domains. Additionally, a file is provided containing a translation between the PDZ domain names used in the test datasets and the PDZ domain names used in the alignment.
(BZ2)

Dataset S4 **Implementation of the predictor of Chen** ***et al.*** **** [**27**] **.** The archive contains data files and python scripts necessary to launch the predictor. The only prerequisite for running the program is an installed python version. Check the README.txt for more information.
(BZ2)

Table S1 **Diversity of amino acids at last five positions of PDZ-binding peptides in the training data of Chen** ***et al.*** **** [**27**] **.**
(PDF)

Table S2 **Filtered numbers of proteins predicted to bind to 1, 2, 3, … or all PDZ domains of MAGI1 (6 PDZs) or SCRIB (4 PDZs).**
(PDF)

Table S3 **Annotations for all proteins tested experimentally in this work for interaction to MAGI1 and SCRIB.** The table contains UniProt IDs and information about biological functions of the proteins with regard to PDZ domain binding as well as published information on interactions with PDZ domain-containing proteins.
(PDF)

Table S4
**Experimental data for all interactions measured.** The table contains “double referenced” and normalised R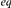 signals obtained for a 10 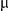
M analyte concentration as well as tentative calculated K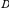 assuming a simple 1∶1 interaction binding isotherm model. These K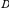 have to be considered with caution, especially for interactions for which weak RU signals were obtained.(PDF)

Text S1 **Recommendations for application of the predictor of Chen** ***et al.*** **** [**27**] **.**
(TXT)
