# Supplementary material for: Putting into Practice Domain-Linear Motif Interaction Predictions for Exploration of Protein Networks
Source: PLoS One. 2011 Nov 1;6(11):e25376. doi: 10.1371/journal.pone.0025376 (PMC3206016; doi:10.1371/journal.pone.0025376)
Supplement: Table S1 — Diversity of amino acids at last five positions of PDZ-binding peptides in the training data of Chen et al. [27] . (PDF) [file pone.0025376.s005.pdf]

Table S1: Diversity of amino acids at five peptide positions in the training data of Chen et al.

ligand pos[a]	observed amino acids																			
0	A	C			F			I		L								V		
-1	A		D	E	F	G	H	I	K	L	M	N	P	Q	R	S	T	V	W	Y
-2	A		D	E	F	G	H	I				N		Q		S	T	V	W	Y
-3	A		D	E	F	G	H	I	K	L		N	P	Q	R	S	T	V	W	Y
-4	A	C	D	E	F	G	H	I	K	L	M	N	P	Q	R	S	T	V		Y

[a]Peptide positions are labelled from 0 to -4 going backwards from the very last amino acid to the fifth last.
