# Supplementary material for: Putting into Practice Domain-Linear Motif Interaction Predictions for Exploration of Protein Networks
Source: PLoS One. 2011 Nov 1;6(11):e25376. doi: 10.1371/journal.pone.0025376 (PMC3206016; doi:10.1371/journal.pone.0025376)
Supplement: Table S2 — Filtered numbers of proteins predicted to bind to 1, 2, 3, … or all PDZ domains of MAGI1 (6 PDZs) or SCRIB (4 PDZs). (PDF) [file pone.0025376.s006.pdf]

Table S2: Filtered numbers of proteins predicted to bind to 1, 2, 3, ... or all PDZ domains of MAGI1 (6 PDZs) or Scribble (4 PDZs)

num. domains	MAGI1	Scribble
1	453	145
2	138	97
3[a]	103	29
4	39	68
5	9	/
6	0	/

[a]e.g. 103 and 29 human proteins were predicted to bind to 3 out of the 6 PDZ domains of MAGI1 and 3 out of the 4 PDZ domains of Scribble, respectively.
