# Supplementary material for: Putting into Practice Domain-Linear Motif Interaction Predictions for Exploration of Protein Networks
Source: PLoS One. 2011 Nov 1;6(11):e25376. doi: 10.1371/journal.pone.0025376 (PMC3206016; doi:10.1371/journal.pone.0025376)
Supplement: Table S3 — Annotations for all proteins tested experimentally in this work for interaction to MAGI1 and SCRIB. The table contains UniProt IDs and information about biological functions of the proteins with regard to PDZ domain binding as well as published information on interactions with PDZ domain-containing proteins. (PDF) [file pone.0025376.s007.pdf]

name	C-terminal sequence	UniprotID	long name	organism	function linked to PDZ	interactions with PDZ-containing proteins				this study
						protein	PDZ	Kd (μM)	source (PMID)	
16E6	SSRTRRETQL	VE6_HPV16	early protein E6	human papilloma virus 16	Binds and targets human PDZ-domain containing proteins to degradation.	MAGI1	2/6	2.5	Fournane et al., 11571640	3, 9 μM
						MAGI1			19285702	
						GOPC	1/1		16878151	
						PTN3	1/1		17166906, 17947517	
						TIP1	1/1		15492812	
						DLG1	2/3		9326658	
						DLG1	3/3		9326658	
						DLG4	1/3		17121805	
						DLG4	2/3		17121805	
						SCRIB			11027293, 19285702, 18160445	✓
TAX1	SEKHFRETEV	TAX_HTL1A	Trans-activating transcriptional regulatory protein of HTLV-1	HTLV-1A	Interaction with PDZ domain-containing proteins induces IL2-independent growth, which may be a factor in multi-step leukemogenesis. Inhibits the action of at least three cellular tumor suppressors TP53/p53, RB1 and DLG1.	DLG1, MAGI3, TIP1			Uniprot	
						MAGI1	2/6	3	Fournane et al.	53 μM
						Erbin			17633453, 19472191	
						SCRIB			18661220	✓
16E6L/V	SSRTRRETQV		early protein E6 **L158V**	human papilloma virus 16		MAGI1	2/6	0.8	Fournane et al., 11571640	1, 2, 18 μM
						DLG1			9326658, 11571640	
						DLG4			17121805	
						CAL	1/1		16878151	
						PTN3	1/1		17947517	

ABC1	QDEKVKESYV	ABCA1_HUMAN	ATP-binding cassette sub-family A member 1	human	cAMP-dependent and sulfonylurea sensitive anion transporter.	SNTA1			14722086	
						SNTB1, SNTB2			16192269, 12054535	
						ARGHEF11, ARGHEF12, DLG2, DLG3, LIN7A, LIN7B, LIN7C, MPDZ			16192269	
NET1	SGGKRKETLV	ARHG8_HUMAN	Neuroepithelial cell-transforming gene 1 protein	human	Acts as guanine nucleotide exchange factor (GEF) for RhoA GTPase.	DLG1, DLG3, DLG4, LIN7C			17938206	
						MAGI1	2/6	5	Fournane et al., 11350080	3 μM
						MAGI1	3/6	no binding	11350080	binding
PTEN	EDQHTQITKV	PTEN_HUMAN	Phosphatidylinositol-3,4,5-trisphosphate 3-phosphatase and dual-specificity protein phosphatase PTEN	human	Modulates cell cycle progression and cell survival, inhibits cell migration and integrin-mediated cell spreading and focal adhesion formation, synapse formation.	MAGI1	3/6		15629897	✓
						MAGI2	3/6		10760291	
						MAGI3	3/6		10748157	
						DLG1, MAST1, MAST2, MAST3			15951562	
VANG2	VMRLQSETSV	VANG2_HUMAN	Vang-like protein 2	human	Plays a role in the regulation of planar cell polarity	SCRIB	3/4		16687519	✓
						SCRIB	34/4		16687519, 16791850	✓
						SCRIB	2/4	weak	16687519	
						SCRIB	4/4	weak	16687519	
						SCRIB	23/4		16791850	
						DVL1, DVL2, DVL3			15456783	
						MAGI3	2/6		15195140	
ADAM17	NRVDSKETEC	ADA17_HUMAN	Disintegrin and metalloproteinase domain-containing protein 17	human	cleaves membrane-anchored and cell-surface proteins for their activation or degradation	DLG1			18930083	
						DLG1	3/3		12668732	
						PTPH1	1/1		12207026	
FZD4	KPGKGSETVV	FZD4_HUMAN	Frizzled-4	human	may be involved in transduction and intercellular transmission of polarity information during tissue morphogenesis and/or in differentiated tissues	MAGI3	2/6		15195140	

GLAST	EKPIDSETKM	EAA1_HUMAN	Excitatory amino acid transporter 1	human	Essential for terminating the postsynaptic action of glutamate by rapidly removing released glutamate from the synaptic cleft.	NHERF1	1/2	17048262	
						NHERF2		20430067	
DLL1	KDECVIATEV	DLL1_HUMAN	Delta-like protein 1	human					
ARHGAP6	NPDALPETLV	RHG06_HUMAN	Rho GTPase-activating protein 6	human	Could regulate the interactions of signaling molecules with the actin cytoskeleton				
TANC1	PKRSFIESNV	TANC1_HUMAN	Tetratricopeptide repeat, ankyrin repeat and coiled-coil domain-containing protein 1	human	may be a scaffold component in the postsynaptic density, interacts probably directly with DLG1 and DLG4				
GLUT7	TASPAKETSF	GTR7_HUMAN	Solute carrier family 2, facilitated glucose transporter member 7	human					
TMEM215	QGRWDHETIV	TM215_HUMAN	Transmembrane protein 215	human					
MARCH3	VKRNSKETVV	MARH3_HUMAN	E3 ubiquitin-protein ligase MARCH3	human	Mutational analyses revealed that the PDZ-binding motif and RING finger are essential for the subcellular localization of MARCH-III and the inhibitory effect on transferrin uptake (16428329), MARCH2 binds PDZs of DLG1 (17980554) but has different C-terminus than MARCH3: LKKVAEETPV				
MAS	CNTVTVETVV	MAS_HUMAN	Proto-oncogene Mas	human	Receptor for angiotensin 1-7, belongs to the G-protein coupled receptor 1 family.				
ATP1A1	GGWVEKETYY	AT1A1_HUMAN	Sodium/potassium-transporting ATPase subunit alpha-1	human					
CYSLTR2	SVWLRKETRV	CLTR2_HUMAN	Cysteinyl leukotriene receptor 2	human					
TTC24	PMESGICTIV	TTC24_HUMAN	Tetratricopeptide repeat protein 24	human					
