# Supplementary material for: Putting into Practice Domain-Linear Motif Interaction Predictions for Exploration of Protein Networks
Source: PLoS One. 2011 Nov 1;6(11):e25376. doi: 10.1371/journal.pone.0025376 (PMC3206016; doi:10.1371/journal.pone.0025376)
Supplement: Table S4 — Experimental data for all interactions measured. The table contains “double referenced” and normalised R signals obtained for a 10 M analyte concentration as well as tentative calculated K assuming a simple 1∶1 interaction binding isotherm model. These K have to be considered with caution, especially for interactions for which weak RU signals were obtained. (PDF) [file pone.0025376.s008.pdf]

BS = bad signal

NM = not measured

MBP-PDZ analyte	peptide	tentative K_D (μM)	RU at 10 μM, 1st exp.	RU at 10 μM, 2nd exp.	norm. RU at 10 μM
MAGI1-2/6	DLL1_05	32	6.4		3.7
MAGI1-3/6	DLL1_05	246	9.8		5.5
SCRIB-3/4	DLL1_05	200	24.8		15.3
SCRIB-34/4	DLL1_05	NM			
SCRIB-4/4	DLL1_05	893	15.1		9.3
MAGI1-2/6	DLL1_10		0.0	0.0	0.0
MAGI1-3/6	DLL1_10	238	13.0		10.0
SCRIB-3/4	DLL1_10	172	42.8		35.9
SCRIB-34/4	DLL1_10	NM			
SCRIB-4/4	DLL1_10	88	16.8		14.1
MAGI1-2/6	16E6_05	7	37.4		24.3
MAGI1-3/6	16E6_05		0.0	0.0	0.0
SCRIB-3/4	16E6_05	12	225.0		156.7
SCRIB-34/4	16E6_05	1	545.0		317.0
SCRIB-4/4	16E6_05		0.0	0.0	0.0
MAGI1-2/6	16E6L/V	2	801.0		520.6
MAGI1-3/6	16E6L/V	69	62.3		39.8
SCRIB-3/4	16E6L/V	18	133.5		93.0
SCRIB-34/4	16E6L/V	3	477.0		277.5
SCRIB-4/4	16E6L/V	47	12.8		8.9
MAGI1-2/6	ABC1_05	91	23.2	21.4	26.6
MAGI1-3/6	ABC1_05	26	88.9	77.1	97.4
SCRIB-3/4	ABC1_05	229	15.0	13.7	18.3
SCRIB-34/4	ABC1_05	10	63.5	55.9	63.7
SCRIB-4/4	ABC1_05		0.0	0.0	0.0
MAGI1-2/6	ARHGAP6_05	2000	10.5	10.3	12.6
MAGI1-3/6	ARHGAP6_05	58	47.0	46.6	55.9
SCRIB-3/4	ARHGAP6_05	216	12.1	12.0	15.7
SCRIB-34/4	ARHGAP6_05	31	40.3	37.2	42.1
SCRIB-4/4	ARHGAP6_05	104	5.0	5.5	6.8
MAGI1-2/6	ARHGAP6_10	18	20.6	18.4	23.7
MAGI1-3/6	ARHGAP6_10	41	54.5	49.9	62.4
SCRIB-3/4	ARHGAP6_10	541	8.9	7.6	10.7
SCRIB-34/4	ARHGAP6_10	28	8.5	6.8	8.3
SCRIB-4/4	ARHGAP6_10		0.0	0.0	0.0
MAGI1-2/6	ABC1_10	45	13.5	12.7	16.2
MAGI1-3/6	ABC1_10	20	92.9	74.0	101.6
SCRIB-3/4	ABC1_10	107	25.8	23.7	32.8
SCRIB-34/4	ABC1_10	8		54.3	60.2
SCRIB-4/4	ABC1_10		0.0	0.0	0.0
MAGI1-2/6	NET1_05	62		60.9	75.4
MAGI1-3/6	NET1_05	23	110.0	101.0	128.5
SCRIB-3/4	NET1_05	308	12.8	21.7	22.9
SCRIB-34/4	NET1_05	15	83.7	76.9	89.0
SCRIB-4/4	NET1_05		0.0	0.0	0.0
MAGI1-2/6	NET1_10	3	394.0	377.0	432.0
MAGI1-3/6	NET1_10	37	67.9		74.8
SCRIB-3/4	NET1_10	217	21.8	34.9	34.1
SCRIB-34/4	NET1_10	63	29.4	27.0	28.3

SCRIB-4/4	NET1_10		0.0	0.0	0.0
MAGI1-2/6	PTEN_05		0.0	0.0	0.0
MAGI1-3/6	PTEN_05	453	25.6	24.0	22.6
SCRIB-3/4	PTEN_05		0.0	0.0	0.0
SCRIB-34/4	PTEN_05		0.0	0.0	0.0
SCRIB-4/4	PTEN_05		0.0	0.0	0.0
MAGI1-2/6	PTEN_10		0.0	0.0	0.0
MAGI1-3/6	PTEN_10	144	25.0		23.3
SCRIB-3/4	PTEN_10		0.0	0.0	0.0
SCRIB-34/4	PTEN_10		0.0	0.0	0.0
SCRIB-4/4	PTEN_10		0.0	0.0	0.0
MAGI1-2/6	TANC1_05		0.0	0.0	0.0
MAGI1-3/6	TANC1_05	14	7.6	6.8	5.8
SCRIB-3/4	TANC1_05	498	24.0	23.0	20.7
SCRIB-34/4	TANC1_05	9	127.0	113.0	88.4
SCRIB-4/4	TANC1_05		0.0	0.0	0.0
MAGI1-2/6	TANC1_10	287	9.8	8.4	10.8
MAGI1-3/6	TANC1_10		0.0	0.0	0.0
SCRIB-3/4	TANC1_10	39	67.8	60.1	81.7
SCRIB-34/4	TANC1_10	2	201.0		214.5
SCRIB-4/4	TANC1_10		0.0	0.0	0.0
MAGI1-2/6	TAX1_05	33	10.7	11.8	14.1
MAGI1-3/6	TAX1_05	75	11.1	11.1	13.6
SCRIB-3/4	TAX1_05	96	32.2	30.3	41.9
SCRIB-34/4	TAX1_05	1	213.0		238.3
SCRIB-4/4	TAX1_05	50	26.9	25.6	35.2
MAGI1-2/6	TAX1_10	53	28.1	25.1	34.9
MAGI1-3/6	TAX1_10	1230	6.5	5.6	7.8
SCRIB-3/4	TAX1_10	29	77.4	67.5	102.0
SCRIB-34/4	TAX1_10		197.0		231.5
SCRIB-4/4	TAX1_10	48	24.2	20.5	31.5
MAGI1-2/6	16E6	3	148.0	134.0	179.7
MAGI1-3/6	16E6		0.0	0.0	0.0
SCRIB-3/4	16E6	18	112.0	103.0	146.8
SCRIB-34/4	16E6	3	143.0	129.0	155.1
SCRIB-4/4	16E6		0.0	0.0	0.0
MAGI1-2/6	GLUT7_05		0.0	0.0	0.0
MAGI1-3/6	GLUT7_05		0.0	0.0	0.0
SCRIB-3/4	GLUT7_05	56	33.8	30.8	44.1
SCRIB-34/4	GLUT7_05	5	89.2	81.7	97.5
SCRIB-4/4	GLUT7_05		0.0	0.0	0.0
MAGI1-2/6	GLUT7_10		0.0	0.0	0.0
MAGI1-3/6	GLUT7_10		0.0	0.0	0.0
SCRIB-3/4	GLUT7_10	45	45.4	40.4	57.5
SCRIB-34/4	GLUT7_10	5	66.5	60.0	70.8
SCRIB-4/4	GLUT7_10		0.0	0.0	0.0
MAGI1-2/6	16E6L/V	18	224.0		277.3
MAGI1-3/6	16E6L/V	76	17.9	19.4	22.7
SCRIB-3/4	16E6L/V	41	33.1	27.1	39.9
SCRIB-34/4	16E6L/V	4	105.0	95.9	111.3
SCRIB-4/4	16E6L/V		0.0	0.0	0.0
MAGI1-2/6	16E6	9	90.7		138.7
MAGI1-3/6	16E6		0.0	0.0	0.0

SCRIB-3/4	16E6	15	126.0	113.0	195.9
SCRIB-34/4	16E6	3	144.0	128.0	186.2
SCRIB-4/4	16E6		0.0	0.0	0.0
MAGI1-2/6	TMEM215_05		5.0		6.1
MAGI1-3/6	TMEM215_05	116	13.2	14.6	16.8
SCRIB-3/4	TMEM215_05	213	5.6		7.4
SCRIB-34/4	TMEM215_05	15	44.4	39.3	45.9
SCRIB-4/4	TMEM215_05		0.0	0.0	0.0
MAGI1-2/6	TMEM215_10		0.0	0.0	0.0
MAGI1-3/6	TMEM215_10	67	20.9	21.6	25.9
SCRIB-3/4	TMEM215_10	128	13.2	11.7	16.5
SCRIB-34/4	TMEM215_10	9	54.4	47.5	56.5
SCRIB-4/4	TMEM215_10		0.0	0.0	0.0
MAGI1-2/6	ADAM17_05		0.0	0.0	0.0
MAGI1-3/6	ADAM17_05		0.0	0.0	0.0
SCRIB-3/4	ADAM17_05		0.0	0.0	0.0
SCRIB-34/4	ADAM17_05		0.0	0.0	0.0
SCRIB-4/4	ADAM17_05		0.0	0.0	0.0
MAGI1-2/6	VANG2_05		0.0	0.0	0.0
MAGI1-3/6	VANG2_05		0.0	0.0	0.0
SCRIB-3/4	VANG2_05	24	31.4	30.4	53.1
SCRIB-34/4	VANG2_05	4	61.7	61.1	88.2
SCRIB-4/4	VANG2_05		0.0	0.0	0.0
MAGI1-2/6	VANG2_10		0.0	0.0	0.0
MAGI1-3/6	VANG2_10		0.0	0.0	0.0
SCRIB-3/4	VANG2_10	12	40.2	38.2	63.7
SCRIB-34/4	VANG2_10	5	50.3	48.4	66.9
SCRIB-4/4	VANG2_10		0.0	0.0	0.0
MAGI1-2/6	ADAM17_10		0.0	0.0	0.0
MAGI1-3/6	ADAM17_10		0.0	0.0	0.0
SCRIB-3/4	ADAM17_10	97		17.6	19.8
SCRIB-34/4	ADAM17_10	BS			
SCRIB-4/4	ADAM17_10		0.0	0.0	0.0
MAGI1-2/6	MARCH3_05	225	12.9	13.5	19.7
MAGI1-3/6	MARCH3_05	BS			
SCRIB-3/4	MARCH3_05	46	9.0	9.7	14.9
SCRIB-34/4	MARCH3_05	7	55.7		74.3
SCRIB-4/4	MARCH3_05		0.0	0.0	0.0
MAGI1-2/6	MARCH3_10	103	36.6	35.3	54.7
MAGI1-3/6	MARCH3_10	686	7.8	6.8	10.9
SCRIB-3/4	MARCH3_10	100	14.4	13.4	22.7
SCRIB-34/4	MARCH3_10	16	65.6	63.8	88.1
SCRIB-4/4	MARCH3_10		0.0	0.0	0.0
MAGI1-2/6	FZD4_05	146	12.3	11.5	19.4
MAGI1-3/6	FZD4_05	BS			
SCRIB-3/4	FZD4_05		0.0	0.0	0.0
SCRIB-34/4	FZD4_05		0.0	0.0	0.0
SCRIB-4/4	FZD4_05		0.0	0.0	0.0
MAGI1-2/6	MAS_05	2740	11.3	10.8	16.7
MAGI1-3/6	MAS_05	90	12.7	12.0	18.4
SCRIB-3/4	MAS_05	158	13.6	13.0	21.5
SCRIB-34/4	MAS_05	16	53.1	51.9	71.0
SCRIB-4/4	MAS_05	433	2.3	2.0	3.5

MAGI1-2/6	MAS_10		0.0	0.0	0.0
MAGI1-3/6	MAS_10	123	6.5	5.9	9.7
SCRIB-3/4	MAS_10	73	14.6	13.6	24.1
SCRIB-34/4	MAS_10	3	64.2	60.8	89.1
SCRIB-4/4	MAS_10		0.0	0.0	0.0
MAGI1-2/6	FZD4_10	23	61.2	56.8	92.0
MAGI1-3/6	FZD4_10		0.0	0.0	0.0
SCRIB-3/4	FZD4_10		0.0	0.0	0.0
SCRIB-34/4	FZD4_10		0.0	0.0	0.0
SCRIB-4/4	FZD4_10		0.0	0.0	0.0
MAGI1-2/6	GLAST_05		0.0	0.0	0.0
MAGI1-3/6	GLAST_05		0.0	0.0	0.0
SCRIB-3/4	GLAST_05	182	7.7	7.6	12.0
SCRIB-34/4	GLAST_05	179	11.6	11.4	15.0
SCRIB-4/4	GLAST_05		0.0	0.0	0.0
MAGI1-2/6	GLAST_10		0.0	0.0	0.0
MAGI1-3/6	GLAST_10		0.0	0.0	0.0
SCRIB-3/4	GLAST_10		0.0	0.0	0.0
SCRIB-34/4	GLAST_10		0.0	0.0	0.0
SCRIB-4/4	GLAST_10		0.0	0.0	0.0
MAGI1-2/6	ADAM17_10		0.0	0.0	0.0
MAGI1-3/6	ADAM17_10		0.0	0.0	0.0
SCRIB-3/4	ADAM17_10	79	33.4	30.7	22.0
SCRIB-34/4	ADAM17_10	8	78.0	76.3	44.3
SCRIB-4/4	ADAM17_10		0.0	0.0	0.0
MAGI1-2/6	ATP1A1_05		0.0	0.0	0.0
MAGI1-3/6	ATP1A1_05		0.0	0.0	0.0
SCRIB-3/4	ATP1A1_05	1280	3.1	3.4	2.5
SCRIB-34/4	ATP1A1_05		0.0	0.0	0.0
SCRIB-4/4	ATP1A1_05		0.0	0.0	0.0
MAGI1-2/6	ATP1A1_10		0.0	0.0	0.0
MAGI1-3/6	ATP1A1_10		0.0	0.0	0.0
SCRIB-3/4	ATP1A1_10	88	5.8	4.9	3.5
SCRIB-34/4	ATP1A1_10		0.0	0.0	0.0
SCRIB-4/4	ATP1A1_10		0.0	0.0	0.0
MAGI1-2/6	CYSLTR2_05	30	68.2	72.9	38.9
MAGI1-3/6	CYSLTR2_05	21	121.0	120.0	65.4
SCRIB-3/4	CYSLTR2_05	103	41.8	40.2	24.2
SCRIB-34/4	CYSLTR2_05	10	278.0	276.0	136.8
SCRIB-4/4	CYSLTR2_05	123	4.0	3.9	2.3
MAGI1-2/6	CYSLTR2_10	4	385.0	370.0	229.3
MAGI1-3/6	CYSLTR2_10	BS			
SCRIB-3/4	CYSLTR2_10	34	153.0	145.0	97.0
SCRIB-34/4	CYSLTR2_10	15	199.0	196.0	107.4
SCRIB-4/4	CYSLTR2_10	202	9.8	9.8	6.4
MAGI1-2/6	TTC24_05	65	11.5	12.9	6.7
MAGI1-3/6	TTC24_05	34	20.2	25.1	12.3
SCRIB-3/4	TTC24_05	239	11.3	11.1	6.6
SCRIB-34/4	TTC24_05	25	87.8	87.2	43.3
SCRIB-4/4	TTC24_05		0.0	0.0	0.0
MAGI1-2/6	16E6L/V	1	475.0	457.0	368.0
MAGI1-3/6	16E6L/V	202	42.3		32.9
SCRIB-3/4	16E6L/V	32	136.0	127.0	111.3

SCRIB-34/4	16E6L/V	4	339.0	317.0	231.8
SCRIB-4/4	16E6L/V	55	11.7	10.3	9.3
MAGI1-2/6	TTC24_10		0.0	0.0	0.0
MAGI1-3/6	TTC24_10	37	20.0	18.5	12.4
SCRIB-3/4	TTC24_10	117	17.1	16.2	11.7
SCRIB-34/4	TTC24_10	49	39.3	39.6	23.1
SCRIB-4/4	TTC24_10		0.0	0.0	0.0
